# Multistrategy Improved Whale Optimization Algorithm and Its Application

**DOI:** 10.1155/2022/3418269

**Published:** 2022-05-27

**Authors:** Lisang Liu, Rongsheng Zhang

**Affiliations:** ^1^School of Electronic, Electrical Engineering and Physics, Fujian University of Technology, Fuzhou, Fujian 350118, China; ^2^National Demonstration Center for Experimental Electronic Information and Electrical Technology Education, Fujian University of Technology, Fuzhou, Fujian 350118, China

## Abstract

To address the shortcomings of the whale optimization algorithm (WOA) in terms of insufficient global search ability and slow convergence speed, a differential evolution chaotic whale optimization algorithm (DECWOA) is proposed in this paper. Firstly, the initial population is generated by introducing the Sine chaos theory at the beginning of the algorithm to increase the population diversity. Secondly, new adaptive inertia weights are introduced into the individual whale position update formula to lay the foundation for the global search and improve the optimization performance of the algorithm. Finally, the differential variance algorithm is fused to improve the global search speed and accuracy of the whale optimization algorithm. The impact of various improvement strategies on the performance of the algorithm is analyzed using different kinds of test functions that are randomly selected. The particle swarm optimization algorithm (PSO), butterfly optimization algorithm (BOA), WOA, chaotic feedback adaptive whale optimization algorithm (CFAWOA), and DECWOA algorithm are compared for the optimal search performance. Experimental simulations are performed using MATLAB software, and the results show that the improved whale optimization algorithm has a better global optimization-seeking capability. The improved whale optimization algorithm is applied to the distribution network fault location of IEEE-33 nodes, and the effectiveness and accuracy of the distribution network fault zone location based on the multistrategy improved whale optimization algorithm is verified.

## 1. Introduction

The swarm intelligence optimization algorithm uses information sharing and competition between populations to find the optimal solution to an objective function by stochastically exploring and exploiting the feasible space with the help of the population evolutionary behavior of organisms. It is increasingly used in the engineering field because of its conceptual simplicity and ease of implementation.

The research on swarm intelligence optimization algorithms has flourished in recent years, and several new algorithms with different mechanisms and superior performance have emerged one after another, for example, the whale optimization algorithm (WOA) [1], monarch butterfly optimization (MBO) [2], slime mould algorithm (SMA) [3], moth search algorithm (MSA) [4], hunger games search (HGS) [5], Runge Kutta method (RUN) [6], colony predation algorithm (CPA) [7], weighted mean of vectors (INFO), [8] and Harris hawks optimization (HHO) [9]. Among them, the whale optimization algorithm was proposed by Seyedali Mirjalili, an Australian academic, in 2016. It is used to search for the optimal solution by simulating the predatory behavior of humpback whales, which has the advantages of simple process and fast convergence. As a new swarm intelligence optimization algorithm, the whale optimized algorithm still has some shortcomings and still has potential for development. It is of great significance to improve the algorithm and expand its application areas [10, 11]. In [12], a chaotic feedback adaptive whale optimization algorithm (CFAWOA) is proposed to improve the shortcomings of low accuracy in finding the best in complex function optimization problems. In [13], a chaotic search strategy-based whale optimization algorithm (CWOA) is proposed, and the algorithm is optimized in the problems of the difficult coordination of exploration and exploitation abilities and falling easily into local optimum. In [14], a Lévy flight-based whale optimization algorithm was used to improve the convergence of the algorithm. An adaptive decision operator-based whale optimization algorithm (IWOA) is proposed in [15], which improves the convergence speed of the algorithm. The continuous improvement in WOA optimization performance has led to WOA being used in a wide range of research areas. Currently, domestic and foreign researchers and scholars have applied the whale optimization algorithm to path planning [16], battery charging [17], optimal reactive power scheduling [18], load prediction [19], fault location [20] and other fields [21].

Scholars have made many improvements and achieved better experimental results at the moment, making WOA optimization relatively mature. However, the whale optimization algorithm still has not completely solved the problem of imbalance between global search ability and local exploitation ability, as well as the problem of easily falling into local optimum. To solve these difficulties encountered in the iterative process of the whale optimization algorithm, this paper analyzes the improvement methods of scholars in recent years, combines the advantages of different improvement methods, and proposes a chaotic whale optimization algorithm that incorporates differential evolution. The improved algorithm is named DECWOA for short in this paper. It is also applied to the distribution network fault location to verify the performance of DECWOA in finding the best performance.

## 2. Whale Optimization Algorithm WOA

**Figure 1:**
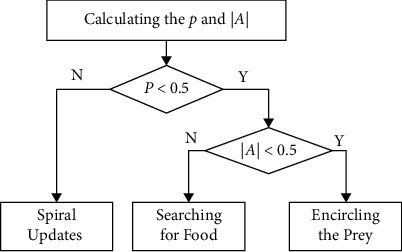
Block diagram of the WOA update mechanism.

This section introduces some preparations before completing the experiments. The optimization principles of WOA are introduced by understanding the three optimization-seeking phases of WOA. The algorithm simulates the predatory behavior of humpback whales and consists of three main stages: searching for food, encircling prey, and swimming spirally to feed. Its selection is determined by a random probability factor *p* ∈ rand[0,1] and a coefficient |*A*|, as shown in Figure 1.

### 2.1. Searching for Food

The food search phase is the process by which whales randomly search for food. The current individual whale randomly selects another individual whale as a target and moves closer to its position. This process corresponds to the global development phase of the algorithm. They should be referred to as the following equation:

(1) $$\begin{array}{c} \left\{\begin{array}{c} X\left(t+1\right)=X_{\text{rand}}\left(t\right)-A\cdot D_{1},\\ D_{1}=\left|C\cdot X_{\text{rand}}\left(t\right)-X\left(t\right)\right|, \end{array}\right. \end{array}$$

where *X*_rand_(*t*) is a randomly selected individual whale from the current whale population, *X*(*t*) is the current individual whale position, *C* is a vector of coefficients randomly distributed between [0,2], and |*A*| should be referred to as the following equation:

(2) $$\begin{array}{c} \left\{\begin{array}{c} A=2a\cdot r-a,\\ a=2-\frac{2t}{T_{\max}}, \end{array}\right. \end{array}$$

where *r* is a random number within [0, 1]. *a* is called the control parameter, *t* is the current number of iterations, and *T*_max_ is the maximum number of iterations. It can be seen that with a decrease linearly from 2 to 0, as the number of iterations *t* increases, the value of the coefficient |*A*| also decreases from 2 to 0.

### 2.2. Encircling the Prey

Whale schools employ a bubble net attack method when feeding on their prey. It consists of two mechanisms, contraction and spiral update, which correspond to the local exploitation phase of the WOA algorithm. In WOA, the individual of the population that has currently obtained the optimal solution is considered to be the target prey and all other individuals move closer to it. The mathematical model of the shrinkage envelope phase should be referred to as the following equation:

(3) $$\begin{array}{c} \left\{\begin{array}{c} X\left(t+1\right)=X_{\text{best}}\left(t\right)-A\cdot D_{2},\\ D_{2}=\left|C\cdot X_{\text{best}}\left(t\right)-X\left(t\right)\right|, \end{array}\right. \end{array}$$

where *X*_best_(*t*) is the best positioned individual whale in the current population and *D*_2_ is the length of the enclosing step. The smaller the value of |*A*|, the smaller the step length of the whale swimming.

### 2.3. Swimming Spirally to Feed

During the spiral renewal phase, other whales will swim in a spiral for food as they approach the optimal whale. It results in a search for the best possible solution between them and the best individual. The initial point of the spiral update is the position of the current whale, and the target end point is the position of the current best whale. The mathematical model can be referred to as the following equation:

(4) $$\begin{array}{c} \left\{\begin{array}{c} X\left(t+1\right)=D_{3}\cdot e^{lb}\cdot \;\cos\left(2\pi l\right)+X_{\text{best}}\left(t\right),\\ D_{3}=\left|X_{\text{best}}\left(t\right)-X\left(t\right)\right|, \end{array}\right. \end{array}$$

where *D*_3_ is the distance between the current individual whale and the best-positioned whale, *b* is a constant coefficient, and *l* belongs to a random number within [0, 1].

## 3. Whale Optimization Algorithm Incorporates Multiple Improvement Strategies

### 3.1. Sine Mapping Population Initialization

The method of population initialization for swarm intelligence optimization algorithms affects the speed of convergence and the accuracy of the algorithm. WOA will use random initial populations in the absence of relevant empirical information, resulting in an inability to ensure that whales are uniformly distributed throughout the solution space. Chaotic mappings generate random sequences from deterministic systems, which are ergodic and stochastic in nature [22, 23]. One-dimensional chaotic mappings, such as logistic mappings and Sine mappings, have simple structures and fast computational speed. In [24], it was verified that Sine chaos had more obvious chaotic properties than logistic chaos. Therefore, Sine chaos is used for the population initialization method of WOA. The expression of Sine chaos self-mapping is as follows:

(5) $$\begin{array}{c} x_{n+1}=\sin\left(\frac{2}{x_{n}}\right),\;n=0,1,\ldots ,N, \end{array}$$

where the initial value *x*_*n*_ cannot be 0, avoiding the immobility and zero point within [−1,1]. At a certain number of iterations, the system output will traverse the entire solution space.

### 3.2. Improved Adaptive Inertia Weights

The inertia weight is an important parameter in WOA. A constant inertia weight will reduce the efficiency of the algorithm and is not conducive to the global optimization of the algorithm [25, 26]. In [27], it is stated that larger inertia weights are beneficial for global optimization. Smaller inertia weights are beneficial for local mining. The ideal inertia weight strategy should present such characteristics: at the beginning of the iteration, it should have larger weights to ensure that the algorithm has a strong global search capability, and at the end of the iteration, it should have smaller weights to ensure that the algorithm has a strong local search capability. Therefore, a reasonable inertia weight is beneficial to balance the global exploration and local exploitation ability of the algorithm.

The improved adaptive inertia weight *ω* is introduced in equations (3) and (4), as shown in the equation.

(6) $$\begin{array}{c} \omega =0.5+\exp\;{\left(\frac{-f_{\text{fit}}\left(x\right)}{u}\right)}^{t}, \end{array}$$

where *f*_fit_(*t*) is the fitness value of whale *x*, *u* is the best fitness value in the whale population at the first iteration of the calculation, and *t* is the current number of iterations. The dynamic nonlinear property of *ω* is used to control the degree of influence of the whale position on the new whale position. The improved update formula is expressed as equations (7) and (8).

(7) $$\begin{array}{c} \left\{\begin{array}{c} X\left(t+1\right)=\omega \cdot X_{\text{best}}\left(t\right)-A\cdot D_{2},\\ D_{2}=\left|C\cdot X_{\text{best}}\left(t\right)-X\left(t\right)\right|, \end{array}\right. \end{array}$$

(8) $$\begin{array}{c} \left\{\begin{array}{c} X\left(t+1\right)=D_{3}\cdot e^{lb}\cdot \;\cos\left(2\pi l\right),\\ W=\omega \cdot X_{\text{best}}\left(t\right),\\ D_{3}=\left|X_{\text{best}}\left(t\right)-X\left(t\right)\right|. \end{array}\right. \end{array}$$

The improved adaptive inertia weights at the beginning of the iteration and the smaller adaptation values ensure that the algorithm has a larger inertia weight. On the contrary, the larger adaptation values at the later stage ensure that the algorithm has a smaller inertia weight, which is beneficial to the global optimization performance of WOA.

### 3.3. Differential Evolutionary Algorithm

The differential evolution algorithm (DE) mainly consists of three processes, namely variation, crossover, and selection [28, 29]. Its control parameters are three, which are the population size, the differential variation parameter *F*, and the crossover probability CR. Firstly, the DE algorithm generates a new generation of variance vectors controlled by the differential variance parameter *F*. Then, the crossover operation between the variance vector and the target vector is performed, and a new trial vector is generated. Finally, greedy selection is performed on the trial vector and the target vector, and the individuals with better fitness are selected to enter the next generation iteration process. After population initialization, three mutually different target vectors *X*_*r*1_, *X*_*r*2_, *X*_*r*3_ are randomly selected in the population, and a new variation vector is generated using the variation factor. It should be referred to as an equation.

(9) $$\begin{array}{c} V_{i}=X_{r1}+F\cdot \left(X_{r2}-X_{r3}\right), \end{array}$$

where *F* is the variance factor, which belongs to the random number within [0,1].

After the mutation operation generates the mutation vector, the crossover operation is performed between the variance vector and the original target vector to generate the test vector. Two common crossover methods are binomial crossover and exponential crossover. Among them, binomial crossover is more commonly used, which is defined as the following equation:

(10) $$\begin{array}{c} U_{i,j}^{\prime }=\left\{\begin{array}{cc} V_{i,j}, & {\text{rand}}_{i,j}\left[0,1\right]\leq \text{CR},\\ x_{i,j}, & \text{otherwise,} \end{array}\right. \end{array}$$

where *V*_*i*,*j*_ is the *j* dimension of the *i* individual generated in the previous step. rand_*i*,*j*_[0,1] is the random number between [0,1]. CR is the crossover factor, which is the random number within [0,1].

After the test vectors are generated, their fitness values are compared with the target vectors. The individual with the better fitness value is selected for the next generation. The *f*_fit_ is the fitness function, and the mathematical model of the selection operation is defined as the following equation:

(11) $$\begin{array}{c} X_{i}\left(t+1\right)=\left\{\begin{array}{cc} U_{i}\left(t\right), & f_{\text{fit}}\left(U_{i}\left(t\right)\right)<f_{\text{fit}}\left(X_{i}\left(t\right)\right),\\ X_{i}\left(t\right), & \text{otherwise.} \end{array}\right. \end{array}$$

The selection process can also be divided into two types: synchronous selection and asynchronous selection. Among them, the asynchronous selection approach has better performance than the synchronous selection approach. In the asynchronous selection approach, after each newly generated test vector is compared with the target vector, the better test vector immediately replaces the corresponding target vector in the population and participates in the update operation of the remaining population individuals. Therefore, the convergence speed of the algorithm is faster.

### 3.4. DECWOA Algorithm Description

The flow of improved DECWOA algorithm is shown as follows:

**Step 1 .**

Set the population size *N*, the solution dimension *D*, the maximum number of iterations *T*_max_, and the current number of iterations *t*.

**Step 2 .**

Introduce Sine mapping to initialize the whale population {*X*_*i*_,  *i*=1,2,…, *N*}. The tabular expression of the Sine chaotic self-mapping is shown in equation (5).

**Step 3 .**

After population initialization, three mutually dissimilar target vectors *X*_*i*1_, *X*_*i*2_, *X*_*i*3_ are randomly selected in the population. A new variation vector is generated using the variation factor, as shown in equation (9).

**Step 4 .**

After the variation operation generates the variation vector, the test vector is generated by crossover operation between the variation vector and the original target vector. The binomial crossover is defined as shown in equation (10).

**Step 5 .**

After the test vectors are generated, their fitness values are compared with those of the target vectors. The individuals with high fitness values are selected to enter the next generation. The mathematical model of the selection operation is defined as shown in equation (11).

**Step 6 .**

Calculate the value of the adaptive inertia weight *ω*. The expression for introducing the new adaptive inertia weights is shown in equation (6).

**Step 7 .**

The fitness value of each whale in the initial state is calculated and ranked by the fitness function.

**Step 8 .**

Refer to the appropriate whale position as the initial optimal solution of the algorithm, the optimal whale position *X*_best_, and its corresponding global optimal fitness value *F*_best_.

**Step 9 .**

Calculate the random probability factor *p* and the coefficient |*A*|. The process set by the whale optimization algorithm is used to determine the next behaviors of the whale and thus selectively update the location of individual whales, as shown in equations (1)–(4).

**Step 10 .**

After the location update is completed, the fitness value is calculated again for all the whale individuals. If *G*_best_^*t*^ ≥ *F*_best_^*t*^, the optimal fitness value *G*_best_^*t*^=*F*_best_^*t*^. Otherwise, keep the current *G*_best_^*t*^ unchanged. *G*_best_^*t*^ is the optimal fitness value in generation *t*.

**Step 11 .**

Determine whether the maximum number of iterations is reached, and if it is satisfied, terminate the iteration and output the current optimal solution. Otherwise, execute Step 9.

## 4. Experimental Results and Analysis

The experimental environment in this paper is based on an Intel(R) Core (TM) i7-7660U processor with 2.50 GHz CPU and 8.00 G of memory. The operating system is Windows 10 (64 bit). The simulation software is MATLAB R2020b. To verify the full performance of DECWOA, the benchmark test functions are selected to contain different spatial features for unimodal and multimodal. The names, exploration intervals, theoretical optimal values, and other attributes of the test functions are shown Table 1. Among them, F1∼F5 are unimodal test functions, F6∼F10 are nonlinear multipeak test functions, and F11∼F14 are solid-dimensional multipeak test functions. The population size of the algorithm is set to 30, and the dimensionality is 30 dimensions. To reduce the random error, the maximum number of iterations is set to 500, and the standard deviation, mean, best value, and worst value of the 30 experiments are obtained.

### 4.1. Time Complexity Analysis of DECWOA

The time complexity of DECWOA is the time consumption of the algorithm, which is the sum of the frequencies of all statements in the algorithm, and it is a function of the size of the problem solved by the algorithm. Considering that factors, such as computer hardware and software, can mask the strengths and weaknesses of the algorithm itself, this paper uses a prior analysis of the estimated algorithm. Assuming that the time complexity of DECWOA depends on the size of the problem, the time complexity *T*(*n*) of the algorithm can be expressed by the following equation:

(12) $$\begin{array}{c} T\left(n\right)=O\left(f\left(n\right)\right), \end{array}$$

where *n* denotes the problem size and *f*(*n*) denotes the number of times the basic operations of the algorithm are repeatedly executed. As the module *n* increases, the growth rate of the time of algorithm execution is proportional to the growth rate of *f*(*n*). Therefore, the smaller *f*(*n*) is, the lower the time complexity of the algorithm and the higher the efficiency of the algorithm.

From a macroscopic point of view, in the optimization process of WOA, assume that the maximum number of iterations of the algorithm is *T*, the dimension is *D*, and the population size is *N*. Then, according to the time complexity formula of the intelligent optimization algorithm, the time complexity of WOA is *O*_1_=*T* × *D* × *N*. For the improved DECWOA, although the number of cycles increases, the structure of the algorithm remains unchanged. It is still determined by the random probability factor *p* and the coefficient |*A*| to determine the foraging mode of individual whales, and the main cycle part of the algorithm is consistent with the basic WOA. Their time complexity is determined by the frequency *f*(*n*) of the innermost statement in the loop with the largest number of nested levels. Therefore, the time complexity of the improved DECWOA can be calculated as *O*_2_=*T* × *D* × *N*. Clearly, *O*_1_=*O*_2_, and there is no increase in time complexity. However, from a microscopic point of view, the time complexity of DECWOA increases to some extent. Each Sine chaos initialization phase did not increase the time complexity. After population initialization, the target vector is selected and the experimental vector is generated using DE. Let the time complexity of DE be *O*_3_=*T*. In a word, from the microscopic point of view, the time complexity of the improved DECW OA is *O*_*t*_=*O*_2_+*O*_3_=*T* × *D* × *N*+*T*, however, the increase in each step does not cause an order of magnitude change. The total time complexity remains *O*_2_=*T* × *D* × *N*.

### 4.2. Impact of Different Improvement Strategies on Algorithm Performance

This section focuses on verifying the reasonableness and superiority of the three improvement strategies, as well as analyzing the magnitude of the contribution of different strategies to the algorithm. Firstly, the basic WOA is defined as WOA-1. The algorithm of WOA incorporating the Sine chaos initialization strategy is defined as WOA-2, the algorithm of introducing a new adaptive inertia weighting strategy into WOA is defined as WOA-3, and the strategy of WOA incorporating the difference variance algorithm is defined as WOA-4. Then, the above four models are compared with DECWOA improved by the hybrid strategy using the benchmark test function for simulation experiments, and the experimental data are shown as Table 2.

As can be seen in Table 2, the algorithm with the addition of the improved strategy has enhanced computational power compared to the basic WOA algorithm. From the test functions F7, F9, F13, and F14, it can be seen that WOA-3 and WOA-4 can find the theoretical optimal value. However, the analysis of the standard deviation of both shows that the optimization ability of WOA-4 is more stable. From the test functions F5, F7, and F11, it can be seen that WOA-1 outperforms WOA-2. From the test function F4, it can be seen that WOA-3 outperforms WOA-4. From the test function F12, it can be seen that WOA-2 outperforms WOA-3. However, the optimal value obtained by WOA-3 in 30 calculations is closer to the theoretical optimal value of F12.

Overall, the three improvement strategies have different degrees of improvement on WOA. The fusion difference evolutionary algorithm improves WOA-4 with the greatest improvement in computational power, followed by WOA-3 with improved inertia weights, and finally WOA-2 with Sine chaos optimized initial population. Therefore, DECWOA is a multistrategy improvement algorithm with fusion difference evolutionary algorithm as the main body and improved inertia weights and fusion chaos theory as the auxiliary means.

### 4.3. Performance Comparison with Other Optimization Algorithms

To verify the effectiveness of the DECWOA algorithm improvements in this paper, this section tests the performance of each algorithm using 14 test functions. To avoid bias in the results because of chance, the algorithms were run 30 times independently on each function. The butterfly optimization algorithm (BOA), the aquila optimizer (AO), the sparrow search algorithm (SSA), the whale optimization algorithm (WOA), the differential evolution (DE), the chaotic feedback adaptive whale optimization algorithm (CFAWOA), and the differential evolutionary chaotic whale optimization algorithm (DECWOA) are listed below on several standard test functions after the experimental results obtained after 30 independent runs (see Table 3).

From the experimental data in Table 3, it can be seen that the calculation accuracy of DECWOA is higher and the development ability is stronger in the single-peak test functions F1∼F5. The standard deviation is the smallest, which proves the good robustness of DECWOA. In the nonlinear multipeak test functions F6∼F10, DECWOA shows the best finding effect. The average value of its optimization search is better than the other six algorithms. In the solid-dimensional multipeak test functions F10 and F12, the average value of DECWOA is inferior to that of SSA and AO algorithms, indicating that the exploration ability of DECWOA still needs to be strengthened. On the whole, the improved DECWOA has greatly improved the merit-seeking ability. For example, in F1∼F14, the basic WOA has less merit-seeking ability than the SSA and AO algorithms. Even though CFAWOA has been improved, its optimization-seeking ability and robustness are not as good as DECWOA proposed in this paper. By comparing DE, WOA, and DECWOA, the experimental data show that the computational accuracy of DE is better than the basic WOA but inferior to DECWOA. Compared with the multipeak test functions F6∼F14, the optimization performance of the improved DECWOA is more prominent in the single-peak test functions F1∼F5.

### 4.4. Convergence Test of DECWOA

**Figure 2:**
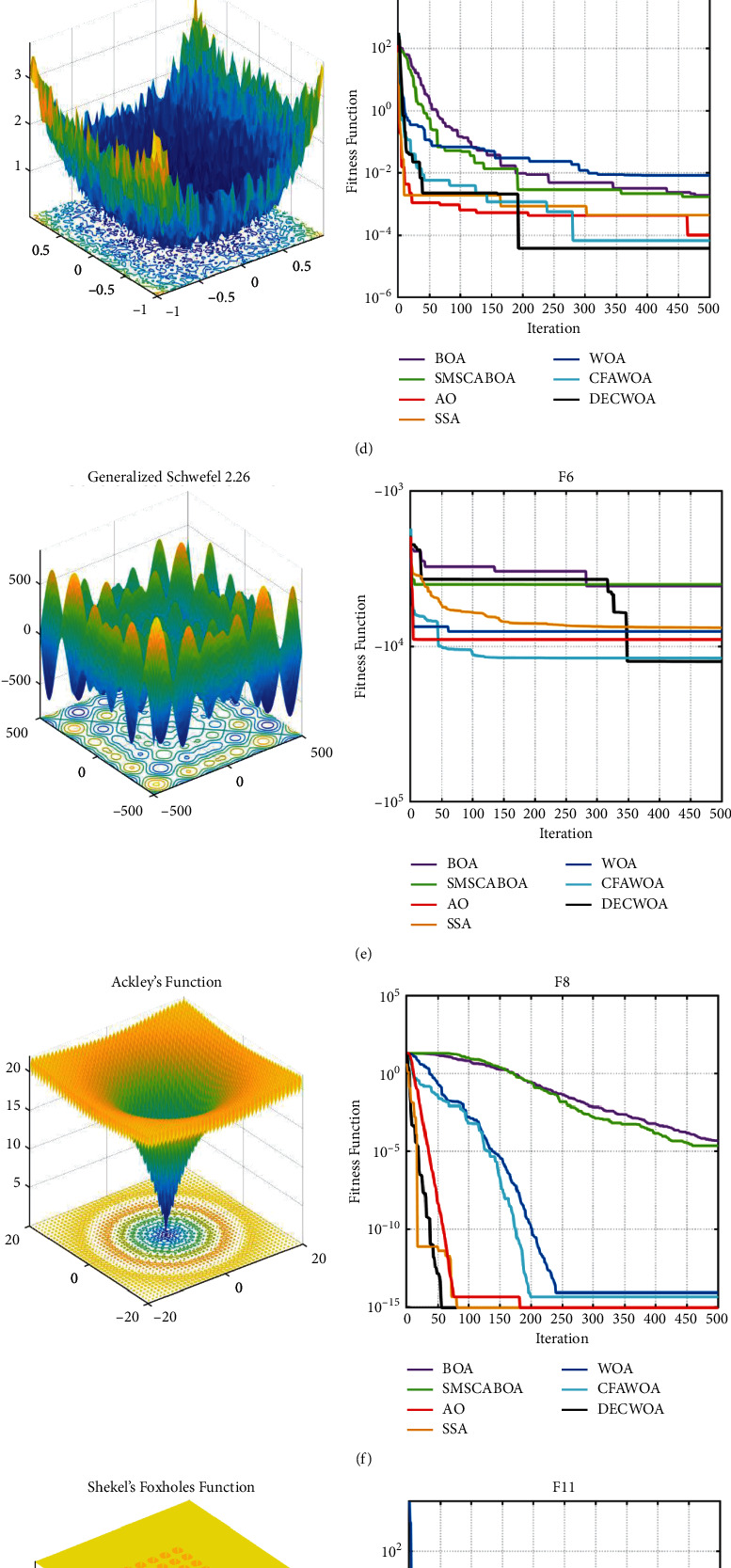
Convergence curves of seven algorithms for fourteen representatives test functions. Note: (a) corresponds to F1, (b) corresponds to F2, (c) corresponds to F3, (d) corresponds to F5, (e) corresponds to F6, (f) corresponds to F8, (g) corresponds to F11, (h) corresponds to F12, and (i) corresponds to F14.

To more intuitively compare the convergence seeking performance of each algorithm in solving the test function, a comparison graph of the convergence curves of the various algorithms mentioned above in the optimization-seeking process is presented. Here, F1∼F3, F5, F6, F8, F11, F12, and F14 are selected, and the function images and convergence curves are shown in Figure 2.

From Figure 2, it can be seen that the improved DECWOA algorithm and algorithm in this paper are highly accurate and have relatively low volatility and good robustness in the process of finding the optimal. It also indirectly confirms that the improved strategy in this paper can make the original WOA jump out of the local optimal state faster. The convergence iteration curves of F1∼F2 show that DECWOA converges approximately linearly and its convergence speed is the fastest. On the convergence iteration curves of F3, F5, and F11, it can be seen that DECWOA converges approximately in a stepwise manner. Compared with WOA and CFAWOA, the convergence speed and the optimization finding accuracy of DECWOA have been significantly improved. The convergence curve of F6 shows that the convergence speed of DECWOA is not as fast as that of WOA and CFAWOA. DECWOA reaches the global optimum in about 350 iterations, while the basic WOA falls into the local optimum after about 60 iterations and does not easily jump out of the local optimum. The convergence iteration curves of F12 and F14 show that DECWOA outperforms the above six algorithms in terms of convergence speed, computational accuracy, and robustness. The convergence iteration curve of F3 shows that WOA basically shows stagnation when it converges to about 50 generations, while the improved DECWOA shows several inflection points before 300 generations, indicating that the improved strategy can make WOA jump out of local optimum effectively. It also indirectly confirms that the strategy of fused differential evolutionary algorithm can make WOA effectively avoid falling into local optimum, and the improved algorithm has good global exploration.

## 5. Application of DECWOA

**Figure 3:**
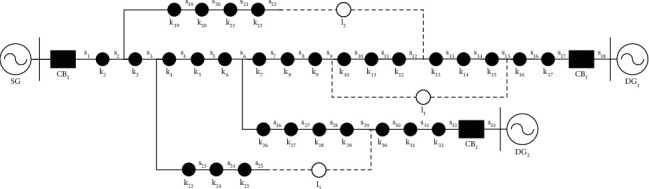
Standard IEEE-33 distribution network topology diagram with DG.

For large-scale optimal solution problems in practical engineering applications, this section applies DECWOA to distribution network fault zone localization. The speed and accuracy of its localization are simulated and experimented. As shown in Figure 3, a standard IEEE-33 distribution network topology diagram containing Distributed Generation (DG) is established. In Figure 3, *CB*_1_ ~ *CB*_2_, *l*_1_ ~ *l*_3_, and *k*_2_ ~ *k*_32_ are switching nodes containing FTU devices, which correspond to the feeder segments from *s*_1_ ~ *s*_33_. *DG*_1_ ~ *DG*_2_ are distributed power sources, and they are selectively put into operation. In this paper, single fault location, multiple fault location, and distortion of fault information are set up for simulation and analysis, respectively. The classical particle swarm optimization (PSO), genetic algorithm (GA), whale optimization algorithm (WOA), and chaotic feedback adaptive whale optimization algorithm (CFAWOA) are selected and compared with the improved DECWOA in the process of fault location in the distribution network. The convergence curves of DECWOA are compared and analyzed. The effectiveness and accuracy of DECWOA in distribution network fault segment localization are verified.

### 5.1. Fault Location Mathematical Model

The fault location in the distribution network is a prerequisite for fault isolation and power supply restoration, which is based on the fault information collected by each feeder terminal unit (FTU) to comprehensively determine the zone where the fault occurs [30, 31]. When a fault occurs in the distribution system, the FTU installed at each switch node is able to detect a current value greater than the pretuned fault current value. The system will upload the fault information to the control master station and then start the fault location procedure for fault location [32, 33].

In the distribution network containing DG, since the tidal direction is not in the unique, this paper defines the direction of the system power pointing to the distributed power and load as the reference direction. Let *I*_*i*_ be the actual measurement state of node switch *i*, and the node is coded in the way shown in the equation.

(13) $$\begin{array}{c} I_{i}=\left\{\begin{array}{c} 1,\text{forward fault current,}\\ 0⟶\text{no fault current,}\\ -1⟶\text{reverse fault current,} \end{array}\right. \end{array}$$

DECWOA can determine the faulty zone using the actual measurement information of the node switches uploaded by the FTU. This process needs to be realized with the help of switching functions. In this paper, the switching function applicable to the multipower distribution network is selected as shown in the equation.

(14) $$\begin{array}{c} I_{i}^{*}=\prod_{i}^{\;d}s_{i\;d}-\prod_{i}^{u}s_{iu}\times \prod_{r}^{n}k_{DGr}, \end{array}$$

where *u* and *d* are the number of all zones upstream and downstream of the node, respectively, *s*_*iu*_ and *s*_*i*  *d*_ are the operating states of the upstream and downstream lines of the node, respectively, *k*_*DGr*_ is the dropout of distributed power *r*, *k*_*DGr*_=1 is the distributed power into the distribution network, and *k*_*DGr*_=0 is the distributed power removed from the distribution network, and *n* is the number of distributed power.

Suppose the actual measured state of the node is A and the desired state calculated by the switching function is B. Then the fitness function represents the difference relationship between A and B. The reasonable construction of this relationship is the key to whether the DECWOA algorithm can accurately achieve fault location in the distribution network. The constructed fitness function is shown in the following equation:

(15) $$\begin{array}{c} F_{\text{fit}}\left(i\right)=\sum_{i=1}^{E}\left|I_{i}-I_{i}^{*}\right|+\mu \sum_{i=1}^{F}\left|s_{i}\right|, \end{array}$$

where *F*_fit_(*i*) is the adaptability value of the node *i*, *E* is the total number of node switches in the distribution system, *μ* is the weighting factor with a value of 0.5, *F* is the total number of zones in the distribution system, and in general, the values of *E* and *F* are equal. The process of distribution network fault location based on intelligent calculation is the process of solving the minimum value of the fitness function.

### 5.2. Experimental Results and Analysis

**Figure 4:**
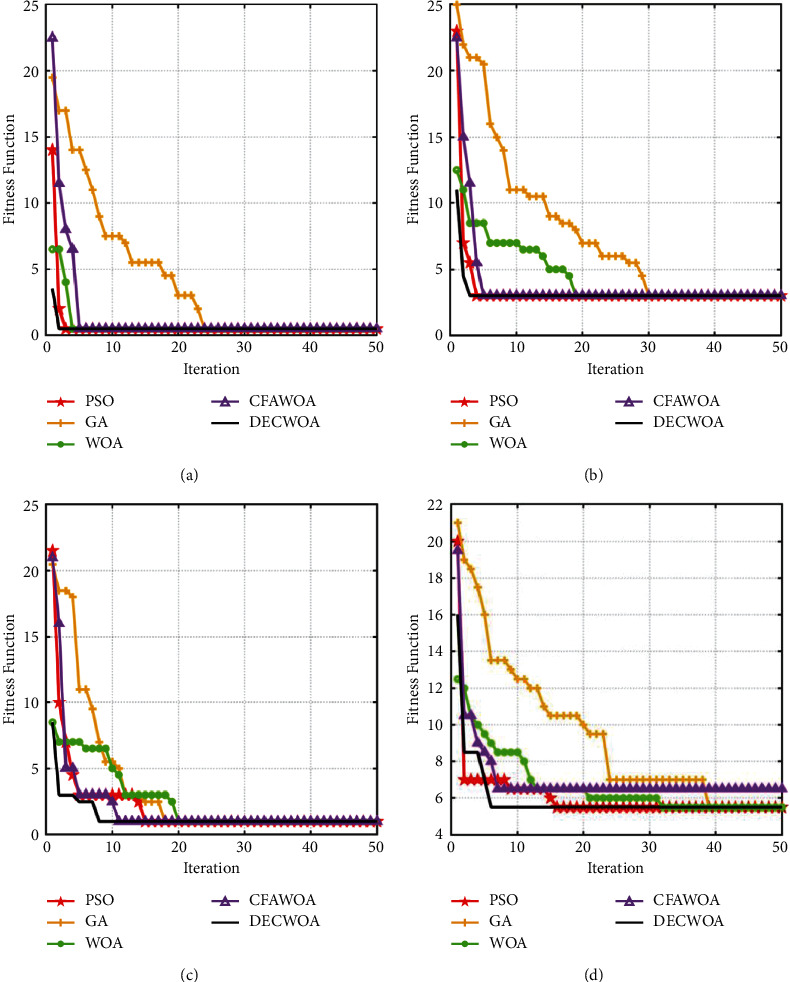
.Comparison of the convergence curves of the five algorithms in the localization process. Note: (a) corresponds to *f*_1_, (b) corresponds to *f*_2_, (c) corresponds to *f*_7_, and (d) corresponds to *f*_8_.

In the simulation experiment, set the population parameter *N*=50 and the maximum number of iterations *T*=50, and the simulation platform is built in MATLAB2021. We set nine different fault types, *f*_1_ ~ *f*_9_. To verify the localization accuracy of each algorithm, the simulation was cycled 50 times for each fault state, and the localization results are shown in Table 4. To more clearly demonstrate the advantages of DECWOA in locating faults in the distribution network with high speed and accuracy, a comparison of the convergence curves of the five algorithms is presented in Figure 4.

From Table 4, it can be seen that in *f*_1_ ~ *f*_5_, when a single fault occurs in the distribution network, all the other four algorithms have high fault location accuracy, except GA, and the fault location accuracy of DECWOA is 100%. The distortion of fault information has no significant effect on the localization accuracy of various algorithms. In *f*_6_ ~ *f*_9_, when multiple faults occurred in the distribution network, the localization accuracy of various algorithms significantly decreased. Among them, GA has the lowest localization accuracy with an average of 92.67%, and DECWOA maintains the highest accuracy with an average of 99.78%. Comparing *f*_1_, *f*_2_ and *f*_6_, *f*_7_, these four sets of data show that the access of DG reduces the accuracy of distribution network fault localization. The accuracy of DECWOA is 98% in *f*_8_ and 100% in other fault conditions. However, in practical engineering, it is less likely that multiple faults will occur in the distribution network at the same time. The improved performance of DECWOA optimization effectively improves its reliability in engineering applications. Compared with the basic WOA, the fault location accuracy of DECWOA is significantly improved.

Figure 4(a) corresponds to *f*_1_, and both distributed power sources are connected to the system operation. In the condition that a single fault occurs in *s*_8_ and the node information is not distorted, all five algorithms can find the minimum adaptation value. Among them, DECWOA is the first to find the optimal fitness value of 0.5, and GA converges the slowest and finally also finds the minimum fitness value of 0.5.  4(b) corresponds to *f*_3_, and both distributed power supplies are connected to the system. In the condition of a single fault at *s*_27_ and information distortion at node *I*_13_, DECWOA is the first to find the optimal adaptation value of 3. However, the convergence of WOA becomes significantly worse.  4(c) corresponds to *f*_7_, in which all five algorithms fall into local optima when multiple faults occur at *s*_12_ and *s*_30_, and no information distortion occurs at nodes. DECWOA is the first to jump out of the local optimum in the 8^th^ iteration and obtains the optimal value of 1.  4(d) corresponds to *f*_8_, in which multiple faults occur at *s*_11_, *s*_20_, and *s*_27_ simultaneously and no information distortion occurs at node *I*_6_. DECWOA is the first to jump out of the local optimum in the 7^th^ iteration and obtain the optimal fitness value of 5.5. The other algorithms take longer time to fall into the local optimum. Among them, CFAWOA fails to jump out of the local optimum, which leads to the fault location error. In conclusion, DECWOA can be well-applied in the distribution network fault location.

## 6. Conclusions

To address the problems of slow convergence and poor global search ability of the underlying WOA in this paper, Sine chaos initialization is used to increase the initial population diversity. The global search and local exploitation ability are improved by introducing a new adaptive inertia weighting strategy. The differential evolution algorithm is also introduced to enhance the ability of the algorithm to jump out of the local optimum. Based on the above multiple improvement strategies, a differential evolution chaotic whale optimization algorithm is proposed. Fourteen benchmark functions and distribution network fault location models are selected for optimization experiments. The results show that the improved whale optimization algorithm based on the hybrid strategy has significantly improved in optimization-seeking accuracy and convergence speed.

## Figures and Tables

**Table 1:** Baseline test functions.

Number	Function's name	Search space	Theoretical best quality
F1	Sphere function	[−100, 100]	0
F2	Schwefel's problem1.2	[−100, 100]	0
F3	Generalized Rosenbrock's function	[−30, 30]	0
F4	Step function	[−100, 100]	0
F5	Quartic function	[−1.28, 1.28]	0
F6	Generalized Schwefel 2.26	[−500, 500]	−12569.5
F7	Generalized Rastrigin's function	[−5.12, 5.12]	0
F8	Ackley's function	[−32, 32]	0
F9	Generalized Griewank's function	[−600, 600]	0
F10	Generalized Penalized function	[−50, 50]	0
F11	Shekel's Foxholes function	[−65.536, 65.536]	1
F12	Kowalik's function	[−5, 5]	0.0003075
F13	Goldstein-Price function	[−2, 2]	3
F14	Hartman's Family	[0, 1]	−3.86

**Table 2:** Comparative experimental results of different improvement strategies.

Function		Experimental comparison			
		WOA-1	WOA-2	WOA-3	WOA-4
F1	Std	4.51559*E* − 08	2.09347*E* − 17	1.84557*E* − 83	0.00000*E* + 00
	Mean	2.79484*E* − 07	1.13353*E* − 17	8.30331*E* − 84	5.23562*E* − 284
	Best	2.29048*E* − 07	5.67353*E* − 21	6.23435*E* − 88	2.03579*E* − 288
	Worst	3.30405*E* − 07	4.84395*E* − 17	4.10483*E* − 83	1.04979*E* − 283

F2	Std	1.70153*E* − 10	1.83991*E* − 10	1.43226*E* − 54	1.11454*E* − 153
	Mean	1.25058*E* − 10	1.15076*E* − 10	7.85979*E* − 55	1.13351*E* − 153
	Best	2.99584*E* − 11	7.22843*E* − 12	4.20392*E* − 60	8.38538*E* − 159
	Worst	4.27458*E* − 10	4.38038*E* − 10	3.30944*E* − 54	2.60495*E* − 153

F3	Std	0.024963674	0.109350354	0.751703418	0.621730369
	Mean	2.89000*E* + 01	2.80000*E* + 01	2.80000*E* + 01	2.63000*E* + 01
	Best	2.88000*E* + 01	2.78000*E* + 01	2.70000*E* + 01	2.50500*E* + 01
	Worst	2.89000*E* + 01	2.81000*E* + 01	2.88000*E* + 01	2.70000*E* + 01

F4	Std	0.555804735	0.374895942	0.007181727	0.016941429
	Mean	4.71000*E* + 00	2.53000*E* + 00	2.93000*E* − 02	5.20688*E* − 02
	Best	4.06000*E* + 00	2.02000*E* + 00	2.20000*E* − 02	3.33000*E* − 02
	Worst	5.29000*E* + 00	2.91000*E* + 00	3.71000*E* − 02	7.27000*E* − 02

F5	Std	0.000538608	0.001572697	0.004444647	9.32354*E* − 05
	Mean	1.642924*E* − 03	3.42854*E* − 03	2.45038*E* − 03	9.90238*E* − 05
	Best	9.46000*E* − 04	1.96257*E* − 03	2.76048*E* − 04	4.03043*E* − 05
	Worst	2.31000*E* − 03	5.85754*E* − 03	1.04037*E* − 02	2.59042*E* − 04

F6	Std	135.48707911	224.1043362	1645.9890973	1583.1336835
	Mean	−3.63000*E* + 03	−4.19242*E* + 03	−1.09483*E* + 04	−1.18032*E* + 04
	Best	−3.82000*E* + 03	−4.52124*E* + 03	−1.26836*E* + 04	−1.26023*E* + 04
	Worst	−3.44000*E* + 03	−3.98124*E* + 03	−8.34938*E* + 03	−9.02012*E* + 03

F7	Std	4.287032*E* − 11	1.66405*E* − 06	0.00000*E* + 00	0.00000*E* + 00
	Mean	3.77032*E* − 11	1.39229*E* − 06	0.00000*E* + 00	0.00000*E* + 00
	Best	3.41023*E* − 13	8.97492*E* − 08	0.00000*E* + 00	0.00000*E* + 00
	Worst	9.76032*E* − 11	4.20305*E* − 06	0.00000*E* + 00	0.00000*E* + 00

F8	Std	5.76251*E* − 06	0.000258291	2.51215*E* − 15	0.00000*E* + 00
	Mean	5.34320*E* − 05	2.03723*E* − 04	4.44423*E* − 15	8.88021*E* − 16
	Best	4.46034*E* − 05	6.71941*E* − 06	8.88423*E* − 16	8.88012*E* − 16
	Worst	6.04323*E* − 05	6.46058*E* − 04	7.99012*E* − 15	8.88135*E* − 16

F9	Std	1.52609*E* − 07	1.55974*E* − 07	0.00000*E* + 00	0.00000*E* + 00
	Mean	2.18044*E* − 07	1.97474*E* − 07	0.00000*E* + 00	0.00000*E* + 00
	Best	7.35032*E* − 08	4.30479*E* − 10	0.00000*E* + 00	0.00000*E* + 00
	Worst	4.20234*E* − 07	4.17068*E* − 07	0.00000*E* + 00	0.00000*E* + 00

F10	Std	0.117121177	0.184990967	0.101764095	0.160370012
	Mean	2.94043*E* + 00	1.67000*E* + 00	2.55000*E* − 01	3.23000*E* − 01
	Best	2.73043*E* + 00	1.47000*E* + 00	1.40000*E* − 01	9.04000*E* − 02
	Worst	2.99034*E* + 00	1.91010*E* + 00	3.62985*E* − 01	5.33000*E* − 01

F11	Std	0.006595458	5.94138*E* − 05	0.887534913	0.00000*E* + 00
	Mean	1.00000*E* + 00	9.98000*E* − 01	1.59000*E* + 00	9.98000*E* − 01
	Best	9.98000*E* − 01	9.98000*E* − 01	9.98000*E* − 01	9.98000*E* − 01
	Worst	1.01000*E* + 00	9.98000*E* − 01	2.98000*E* + 00	9.98000*E* − 01

F12	Std	9.35683*E* − 05	9.72888*E* − 06	0.000797524	3.85761*E* − 05
	Mean	5.01044*E* − 04	3.27153*E* − 04	7.73325*E* − 04	3.29173*E* − 04
	Best	3.63403*E* − 04	3.16032*E* − 04	3.11320*E* − 04	3.09163*E* − 04
	Worst	5.83340*E* − 04	3.36011*E* − 04	2.18264*E* − 03	3.97427*E* − 04

F13	Std	0.141889101	0.000134211	4.47202*E* − 05	0.000207401
	Mean	3.13232*E* + 00	3.00000*E* + 00	3.00000*E* + 00	3.00000*E* + 00
	Best	3.00003*E* + 00	3.00000*E* + 00	3.00000*E* + 00	3.00000*E* + 00
	Worst	3.30237*E* + 00	3.00000*E* + 00	3.00000*E* + 00	3.00000*E* + 00

F14	Std	0.072140138	0.001317012	0.010294048	0.000532011
	Mean	−3.76034*E* + 00	−3.8600*E* + 00	−3.86000*E* + 00	−3.86000*E* + 00
	Best	−3.86043*E* + 00	−3.8600*E* + 00	−3.86000*E* + 00	−3.86000*E* + 00
	Worst	−3.67001*E* + 00	−3.8600*E* + 00	−3.84000*E* + 00	−3.86000*E* + 00

**Table 3:** Comparative experimental results of different optimization algorithms.

Function		Experimental Comparison						
		BOA	AO	SSA	WOA	CFAWOA	DE	DECWOA
F1	Std	1.15689*E* − 07	7.17623*E* − 129	5.76465*E* − 82	1.09694*E* − 77	0.00000*E* + 00	1.10035*E* − 227	0.00000*E* + 00
	Mean	3.75375*E* − 07	3.59224*E* − 129	2.88294*E* − 82	1.03964*E* − 77	1.50843*E* − 199	2.65158*E* − 177	7.57439*E* − 281
	Best	2.63837*E* − 07	5.76183*E* − 150	0.00000*E* + 00	1.99046*E* − 82	9.43048*E* − 204	2.6923*E* − 199	1.24974*E* − 284
	Worst	4.93932*E* − 07	1.44945*E* − 128	1.15446*E* − 81	2.32947*E* − 77	5.38249*E* − 199	7.95564*E* − 177	2.40433*E* − 280

F2	Std	3.78976*E* − 10	8.84757*E* − 75	6.85326*E* − 46	1.74286*E* − 54	1.14893*E* − 122	1.83797*E* − 107	2.75246*E* − 150
	Mean	3.02274*E* − 10	6.71386*E* − 75	3.43464*E* − 46	8.76034*E* − 55	6.79042*E* − 123	1.29946*E* − 107	1.38049*E* − 150
	Best	2.23027*E* − 14	1.24559*E* − 77	0.0000*E* + 00	2.14797*E* − 57	3.43868*E* − 126	1.71259*E* − 126	2.59043*E* − 155
	Worst	8.00049*E* − 10	1.82578*E* − 74	1.37856*E* − 45	3.49578*E* − 54	2.39786*E* − 122	3.89803*E* − 107	5.54661*E* − 150

F3	Std	1.46819*E* − 07	4.82826*E* − 143	1.33095*E* − 97	3.63932*E* − 56	5.02996*E* − 130	1.70936*E* − 133	0.00000*E* + 00
	Mean	3.42043*E* − 07	4.84945*E* − 143	6.65032*E* − 98	4.81636*E* + 04	2.59746*E* − 130	1.22267*E* − 133	6.97643*E* − 195
	Best	2.36235*E* − 07	1.29869*E* − 149	9.49000*E* − 149	3.25000*E* + 04	1.86978*E* − 135	9.38623*E* − 156	2.03225*E* − 204
	Worst	5.58263*E* − 07	1.10898*E* − 142	2.66000*E* − 97	5.50000*E* + 04	1.01958*E* − 129	3.64865*E* − 133	2.77642*E* − 194

F4	Std	1.45389*E* − 05	2.51446*E* − 57	2.75065*E* − 46	26.26153745	1.33868*E* − 102	6.14682*E* − 103	2.26475*E* − 135
	Mean	7.62368*E* − 05	1.26467*E* − 57	1.38455*E* − 46	2.71000*E* + 01	6.70849*E* − 103	4.70798*E* − 103	1.13954*E* − 135
	Best	5.81373*E* − 05	4.15866*E* − 80	0.00000*E* + 00	1.89000*E* + 00	4.40275*E* − 107	0.00000*E* + 00	9.25859*E* − 139
	Worst	9.16575*E* − 05	5.03978*E* − 57	5.50768*E* − 46	5.59000*E* + 01	2.68957*E* − 102	1.33957*E* − 102	4.53684*E* − 135

F5	Std	0.000304159	0.000129661	0.000371841	0.001796123	1.12746*E* − 05	2.38558*E* − 05	6.25793*E* − 05
	Mean	1.70355*E* − 03	1.44979*E* − 04	4.59000*E* − 04	1.61000*E* − 03	4.57038*E* − 05	6.40005*E* − 05	7.08953*E* − 05
	Best	1.39358*E* − 03	7.107979*E* − 05	1.27000*E* − 04	2.02000*E* − 04	3.42012*E* − 05	3.62615*E* − 05	2.16964*E* − 05
	Worst	2.05245*E* − 03	3.39456*E* − 04	9.32000*E* − 04	4.24000*E* − 03	6.12065*E* − 05	9.45679*E* − 05	1.61964*E* − 04

F6	Std	340.3553381	4159.246398	1334.570924	1829.634873	2634.429438	1279.258654	1795.295025
	Mean	−3.54224*E* + 03	−1.05375*E* + 04	−7.59000*E* + 03	−1.13000*E* + 04	−1.13375*E* + 04	−1.19745*E* + 04	−1.16957*E* + 04
	Best	−3.91246*E* + 03	−1.26278*E* + 04	−9.57000*E* + 03	−1.26000*E* + 04	−1.26276*E* + 04	−1.28452*E* + 04	−1.26857*E* + 04
	Worst	−3.09146*E* + 03	−4.21386*E* + 03	−6.70000*E* + 03	−8.64000*E* + 03	−7.30859*E* + 03	−8.64000*E* + 03	−8.02895*E* + 03

F7	Std	2.84157*E* − 10	0.00000*E* + 00	0.00000*E* + 00	0.00000*E* + 00	0.00000*E* + 00	0.00000*E* + 00	0.00000*E* + 00
	Mean	1.60364*E* − 10	0.00000*E* + 00	0.00000*E* + 00	0.00000*E* + 00	0.00000*E* + 00	0.00000*E* + 00	0.00000*E* + 00
	Best	2.33275*E* − 12	0.00000*E* + 00	0.00000*E* + 00	0.00000*E* + 00	0.00000*E* + 00	0.00000*E* + 00	0.00000*E* + 00
	Worst	5.85463*E* − 10	0.00000*E* + 00	0.00000*E* + 00	0.00000*E* + 00	0.00000*E* + 00	0.00000*E* + 00	0.00000*E* + 00

F8	Std	9.99847*E* − 06	0.00000*E* + 00	0.00000*E* + 00	3.52342*E* − 15	2.05116*E* − 15	1.45039*E* − 15	1.77636*E* − 15
	Mean	5.51047*E* − 05	8.88570*E* − 16	8.88000*E* − 16	3.44585*E* − 15	2.66796*E* − 15	2.66454*E* − 15	1.78648*E* − 16
	Best	4.51607*E* − 05	8.80000*E* − 16	8.88000*E* − 16	4.41738*E* − 16	8.88075*E* − 16	8.88618*E* − 16	3.88357*E* − 16
	Worst	6.89840*E* − 05	8.88093*E* − 16	8.88000*E* − 16	7.99965*E* − 15	4.44796*E* − 15	4.44069*E* − 15	4.44686*E* − 15

F9	Std	1.46303*E* − 07	0.00000*E* + 00	0.00000*E* + 00	0.043835506	0.00000*E* + 00	0.00000*E* + 00	0.00000*E* + 00
	Mean	2.09702*E* − 07	0.00000*E* + 00	0.00000*E* + 00	2.19000*E* − 02	0.00000*E* + 00	0.00000*E* + 00	0.00000*E* + 00
	Best	7.93603*E* − 08	0.00000*E* + 00	0.00000*E* + 00	0.00000*E* + 00	0.00000*E* + 00	0.00000*E* + 00	0.00000*E* + 00
	Worst	3.66570*E* − 07	0.00000*E* + 00	0.00000*E* + 00	8.77000*E* − 02	0.00000*E* + 00	0.00000*E* + 00	0.00000*E* + 00

F10	Std	0.001515476	6.19254*E* − 05	2.78732*E* − 07	0.314675773	0.344948649	0.04999952	0.172051704
	Mean	2.99030*E* + 00	4.72000*E* − 05	3.09759*E* − 07	4.29000*E* − 01	7.77000*E* − 01	9.62645*E* − 01	3.38000*E* − 01
	Best	2.99000*E* + 00	1.81000*E* − 06	2.26556*E* − 08	1.15000*E* − 01	4.21000*E* − 01	3.06721*E* − 01	1.49000*E* − 01
	Worst	2.99000*E* + 00	1.33000*E* − 04	5.85685*E* − 07	8.49000*E* − 01	1.25000*E* + 00	6.9980*E* − 01	5.34000*E* − 01

F11	Std	0.012411974	0.949646322	5.8362500000	4.647013825	0.00000*E* + 00	4.603359426	0.00000*E* + 00
	Mean	1.00000*E* + 00	2.24000*E* + 00	9.75000*E* + 00	3.94467*E* + 00	9.98000*E* − 01	4.253066667	9.98000*E* − 01
	Best	9.98000*E* − 01	9.98000*E* − 01	9.98000*E* − 01	9.98876*E* − 01	9.98000*E* − 01	9.98000*E* − 01	9.98000*E* − 01
	Worst	1.02000*E* + 00	2.98000*E* + 00	1.27000*E* + 01	1.08869*E* + 01	9.98000*E* − 01	1.07632*E* + 01	9.98000*E* − 01

F12	Std	4.23456*E* − 05	0.000135416	4.46346*E* − 07	0.000738262	0.000201337	3.67346*E* − 05	8.69472*E* − 06
	Mean	5.06000*E* − 04	5.94000*E* − 04	3.08000*E* − 04	1.32000*E* − 03	5.67000*E* − 04	0.000348965	3.20538*E* − 04
	Best	4.67000*E* − 04	3.97000*E* − 04	3.08000*E* − 04	3.87000*E* − 04	3.62000*E* − 04	0.000307718	3.13043*E* − 04
	Worst	5.51000*E* − 04	6.98000*E* − 04	3.08000*E* − 04	2.16000*E* − 03	7.42000*E* − 04	0.000396938	3.31835*E* − 04

F13	Std	0.091946882	0.021166877	0.00011547	0.000163299	0.00000*E* + 00	0.00000*E* + 00	0.000150000
	Mean	3.08000*E* + 00	3.01000*E* + 00	3.00000*E* + 00	3.00000*E* + 00	3.00000*E* + 00	3.00000*E* + 00	3.00000*E* + 00
	Best	3.01063*E* + 00	3.00000*E* + 00	3.00000*E* + 00	3.00000*E* + 00	3.00000*E* + 00	3.00000*E* + 00	3.00000*E* + 00
	Worst	3.21969*E* + 00	3.05000*E* + 00	3.00000*E* + 00	3.00000*E* + 00	3.00000*E* + 00	3.00000*E* + 00	3.00000*E* + 00

F14	Std	0.366127815	0.001438749	0.00000*E* + 00	0.003880185	0.000141421	0.000164992	0.000732575
	Mean	−3.86268*E* + 00	−3.86268*E* + 00	−3.86268*E* + 00	−3.86268*E* + 00	−3.86268*E* + 00	−3.86268*E* + 00	−3.86268*E* + 00
	Best	−3.83954*E* + 00	−3.86267*E* + 00	−3.86268*E* + 00	−3.86268*E* + 00	−3.86268*E* + 00	−3.86268*E* + 00	−3.86268*E* + 00
	Worst	−3.89554*E* + 00	−3.86467*E* + 00	−3.86268*E* + 00	−3.86268*E* + 00	−3.86268*E* + 00	−3.86268*E* + 00	−3.86268*E* + 00

**Table 4:** Fault location results based on various algorithms.

Number	Faulted sections	Distortion information	DG status	Accuracy (%)				
				PSO	GA	WOA	CFAWOA	DECWOA
*f*_1_	S8	No	[1, 1]	98	94	98	100	100
*f*_2_	S27	I13	[1, 1]	98	94	100	98	100
*f*_3_	S9	I4	[1, 0]	100	94	98	96	100
*f*_4_	S18	No	[1, 0]	100	96	98	98	100
*f*_5_	S13	I3, I17	[0, 1]	98	94	96	98	100
*f*_6_	S15, S26	I7, I20	[0, 1]	98	94	98	96	100
*f*_7_	S12, S30	I16	[1, 1]	96	92	96	96	100
*f*_8_	S11, S20, S27	I5, I17	[1, 0]	94	88	90	94	98
*f*_9_	S10, S21, S25, S31	No	[0, 1]	92	88	92	94	100

## Data Availability

Some data of our team need to be kept confidential. If necessary, please ask the authors for it.
